# Editorial: Ionizing radiation and reproductive health

**DOI:** 10.3389/fpubh.2023.1147934

**Published:** 2023-02-07

**Authors:** Hosam M. Saleh, Amal I. Hassan

**Affiliations:** Department of Radioisotope, Nuclear Research Center, Egyptian Atomic Energy Authority, Cairo, Egypt

**Keywords:** ionizing radiation, cancer treatment adjustment, cancer diagnosis, radiation protection, sustainable materials for buildings

Humans are continually vulnerable to different types of radiation, whether from natural sources such as sunshine or fabricated devices. Ionizing radiation is crucial in human existence, such as the sources utilized in medical diagnostics, such as X-rays and computed tomography scans (CTs) (1). Most hospitals across the world utilize radiation for cancer diagnosis or treatment. While this imaging technique offers benefits, the potential cancer risks associated with these studies remain controversial. Radiation in the medical community with the concept of saturation (2). There can be a state of equilibrium. Ionizing radiation (IR) industrial applications, including foodstuffs irradiation and decontamination of medical and other instruments, play a significant role in our daily lives (3).

Although a cell can usually heal itself, damage to its fundamental DNA and repair systems can render it unable to operate normally. Cells die and the mucous membrane sloughs off. It is thought that a high radiation dosage delivered in several doses over a short period is better for cell healing than protracted low-dose exposure (1).

Normal tissue damage occurs because of both therapeutic and unintentional ionizing radiation exposure (4). Ionizing radiation causes cell damage and is connected with teratogenic consequences in humans. Direct radiation, particularly to the fetus in the early stages of development (first trimester), is teratogenic and can result in fetal mortality. According to studies, radiation exposure increases the risk of acquiring cancer, and the risk increases with the dosage, thus the higher the dose, the greater the risk (5). Children have a greater lifetime likelihood of cancer mortality from CTs than adults, and the risk is thought to be directly connected to an increased incidence of solid tumors, leukemia, lymphomas, and myelodysplasias (6).

Internal exposition the assessment of occupational exposure to integrated radionuclides is fraught with large uncertainties, which are frequently bigger than those associated with external radiation are. Ionizing radiation has a gonadal toxic effect, which can lead to ovarian insufficiency, pubertal arrest, and eventually infertility (7). Radiation protection aims to reduce unneeded radiation exposure in order to minimize the serious repercussions of ionizing radiation.

The development of harmful radiation protection agents has been a subject of investigation for decades. However, effective (ideal) radiation detectors and radiation modulators remain an unsolved problem. Natural materials can be utilized as natural radiosensitizers exhibiting results in radiotherapy cancer therapies while decreasing IR harm to healthy cells and tissues (8, 9).

Therefore, this Research Topic entitled “*Ionizing radiation and reproductive health*” has been proposed with the aim of bringing together studies relevant to human health and radiation risks, such as cancer or non-cancer risks associated with radiation exposure, together with recent views on innovative strategies for protection. Based on the foregoing, the topic of this issue consists of four papers in which a group of researchers in different disciplines participated. Below is a brief summary of these papers. Tremendous advances have been made in radiation protection methods. Among these, Yuan et al. reported that disulfiram is one method used to protect against radiation-induced intestinal infections. In this study, rats were used as a model in which intestinal injury was induced after exposure to high doses of radiation. This work provides clear evidence of the potential efficacy of disulfiram against intestinal injury in both radiotherapy and accidental exposure. The results of this research revealed that disulfiram reduces the accumulation of DNA damage and promotes the survival of intestinal stem cells by affecting the cell cycle after irradiation. Elshami et al. demonstrate an acceptable quality dose for adult patients succumbing CTs (head, chest, and abdomen). This study was conducted in four major hospitals of the Ministry of Health and Prevention in the United Arab Emirates (UAE). The results obtained could be beneficial of optimizing dosage and diagnostics reliability, which can enhance the confidence of specialists. Zhao et al. develop an assay for estimation of low-level infrared exposure based on mass spectrometry quantification of H-H2AX in blood. *In vitro* cytodynamic monitoring experiments show that DNA damage occurred rapidly and then repaired slowly over the course of the post-irradiation period even after exposure to very low doses of IR. These findings show that this test has the potential to be useful in radiation biometry and environmental hazard identifications. Shubayr and Alashban determine doses of organs in the uterus and prostate and evaluating the lifetime risk of cancer and mortality resulting from examinations. This study called for efforts to reduce patient doses while maintaining image quality. It also recommended the improvement of various CT protocols for treatment planning to reduce radiation dose.

Together, the articles in this special issue provide a clear example of the harms of radiation to human health in an attempt to focus on efforts to avoid such harms and help in finding possible solutions. Above all, with taking into consideration the wisdom that says prevention is better than cure, we must highlight technological advancements in the field of radiation protection, such as the development of sustainable and cost-effective materials for use in the construction of radiation shields (10, 11), the development of environmentally friendly methods for treating radioactive waste (12, 13), and the improving the characterization of the materials used in immobilizing (14, 15) or packaging of radioactive waste to attain human-safe and clean environment (16).

We hope that readers will find these papers interesting and beneficial in their innovative research in this field. The Editors of this Research Topic are like to express their appreciation to all of the authors for their significant contributions, as well as to the professional reviewers for their time, devotion, and valuable comments.

## Author contributions

All authors contributed equally to the conception, drafting, and editing of this editorial manuscript, moreover, they contributed equally to guest editing the Research Topic.
